# When Innate Immunity Meets Angiogenesis—The Role of Toll-Like Receptors in Endothelial Cells and Pulmonary Hypertension

**DOI:** 10.3389/fmed.2020.00352

**Published:** 2020-07-31

**Authors:** Aneel Bhagwani, A. A. Roger Thompson, Laszlo Farkas

**Affiliations:** ^1^Division of Pulmonary, Critical Care and Sleep Medicine, Department of Internal Medicine, Davis Heart & Lung Research Institute, The Ohio State University, Columbus, OH, United States; ^2^Department of Physiology and Biophysics, Virginia Commonwealth University, Richmond, VA, United States; ^3^Department of Infection, Immunity & Cardiovascular Disease, Faculty of Medicine, Dentistry & Health, University of Sheffield, Sheffield, United Kingdom

**Keywords:** toll-like receptors, endothelial cells, angiogenesis, pulmonary hypertension, immunology

## Abstract

Toll-like receptors serve a central role in innate immunity, but they can also modulate cell function in various non-immune cell types including endothelial cells. Endothelial cells are necessary for the organized function of the vascular system, and part of their fundamental role is also the regulation of immune function and inflammation. In this review, we summarize the current knowledge of how Toll-like receptors contribute to the immune and non-immune functions of the endothelial cells.

## Toll-Like Receptors

The human body is constantly exposed to exogenous immunological triggers and reacts to these triggers after recognizing their associated molecules. Janeway proposed over 20 years ago that invading microorganisms, such as viruses and bacteria, have specific molecular patterns, so-called pathogen-associated molecular patterns (PAMPs), that trigger recognition by the immune system and named these pattern recognition receptors (PRRs) (1). The PAMPs are sensed by germline-encoded evolutionary conserved host sensors called pathogen recognition receptors or PRRs and form a key element of the innate immune system (2, 3). PRRs are not only present in typical immune cells, such as monocytes, macrophages, and T lymphocytes, but also are expressed in non-immune cells, including endothelial cells (4). Four classes of PRRs are known today: (1) the Toll-like receptors (TLRs) which we will focus on in this review; (2) C-type lectin receptors (CLRs): this large family of cell surface transmembrane receptors bind carbohydrates via specific recognition domains and are important for the immune response to fungal pathogens (5); (3) retinoic acid-inducible gene-I (RIG-I)-like receptors (RLR) which are cytoplasmic sensors of viral RNA or self-processed RNA (6, 7); and (4) nucleotide-binding oligomerization domain (NOD)-like receptors (NLRs) which are cytoplasmic receptors that recognize PAMPs. In contrast to TLRs, NLRs mainly signal via the formation of a multimeric protein complex called the inflammasome (8). Of these four receptor families, TLRs are the most extensively studied class. Although TLRs are an integral part of the innate immune system, their expression is not limited to immune cells but their presence can also be detected in non-immune cells such as endothelial cells, which are further discussed in this review article. The name “Toll-like receptor” originates from the structural homology of TLRs with the Toll transmembrane protein which is important for embryonic development in *Drosophila melanogaster* (9). Toll has an important role for antibacterial defense in Drosophila (10), because mutations in the Toll gene decrease the antimicrobial and antifungal response, leading to increased susceptibility to infection and death (11). The cytoplasmic domain of the Toll protein shares homology with human interleukin-1 (IL-1) receptors (IL-1Rs), leading to similar biochemical signal transduction by IL-1Rs and Toll protein (12). Human transmembrane receptors with structural homology to the Drosophila Toll protein were categorized as Toll-like receptors (TLRs) (13). At present, 10 TLRs have been identified in humans (TLR1–10) and 12 (TLR1–9, 11, and 13) in mice (14). TLRs 1–9 are highly conserved in mammals, TLR10 is non-functional in mice, and the human TLR11 gene contains a stop codon that results in lack of production of functional TLR11 (15, 16). The TLRs are localized to either the cell surface membrane or the membranes of intracellular compartments. TLRs 1, 2, 4, 5, 6, and 11 are found at the cell surface and detect extracellular PAMPs, whereas TLR 3, 7, 8, and 9 bind intracellular PAMPs localized to intracellular vesicles such as endosomes and lysosomes, or vesicles derived from the endoplasmic reticulum (17).

In addition to recognizing extracellular and intracellular PAMPs, the immune system plays an important role in the response to non-pathogenic conditions, such as trauma, ischemia, and autoimmune disorders. Polly Matzinger proposed a danger signal model paralleling the concept of PAMPs and suggested that any molecule that is normally not secreted from the cell could activate an immune response if released from the cell in response to injury (18). This damage model further evolved to the concept of damage-associated molecular patterns (DAMPs) (19). Similar to the recognition of PAMPs by PRRs, certain PRRs, including TLR2 and TLR4, bind DAMPs (20). A list of the most common PAMPs (pathogen-associated molecules) and DAMPs (self-molecules) that activate the different TLRs is shown in Table 1.

**Table 1:** Ligands for various toll like receptors.

**TLR**	**Ligand**	
	**PAMP**	**DAMP**
TLR1	Bacterial lipoproteins (21, 22)	
TLR2	Soluble peptidoglycan (SPGN) (23)	Biglycan (24)
	Lipoteichoic acid (LTA) (23, 25)	High Mobility Group Box 1 HMGB1(26)
	Pam_3_CSK_4_ (27)	Monosodium urate crystals (28, 29)
		Calcium Pyrophosphate Dihydrate (28)
		Human cardiac myosin and C0C1f fragment of cardiac myosin binding protein-C (30, 31)
TLR3	Viral dsRNA and Polyinosinic:polycytidylic acid (poly(IC)) (32)	dsRNA from necrotic cells (33)
	siRNA (34)	mRNA (35),
TLR4	Lipopolysaccharide LPS (36)	Biglycan (24)
		High-mobility group box 1 (HMGB1) (26)
		Fibrinogen (37)
		Heparan sulfate (38, 39)
		C0C1f fragment of cardiac myosin binding protein-C (30)
TLR5	Flagellin (40)	
TLR6	Diacylated lipoproteins (41)	
TLR7	Guanosine and uridine-containing ssRNA (42)	Human cardiac myosin (31)
TLR8	Single-stranded RNA (ssRNA), bacterial RNA (43–45)	
TLR9	Unmethylated CpG oligodinucleotides (ODNs) from bacterial DNA (46)	Mitochondrial CpG-ODN (47)
TLR10	Human immunization virus-1 (HIV-1) proteins (48)	

*Ligands for various Toll-like receptors differentiated into PAMPs and DAMPs. PAMP, pathogen-associated molecular patterns; DAMP, damage-associated molecular patterns; dsRNA, double-stranded RNA; ssRNA, single-stranded RNA; ODN, oligodinucleotides; mRNA, messenger RNA*.

## Toll-Like Receptor Signaling

TLRs are evolutionary conserved type 1 transmembrane glycoprotein receptors with an ectodomain and a cytosolic domain. The ectodomain contains varying numbers of leucine-rich repeats (LRRs) that are required for ligand binding. Vertebrate TLRs contain 16–28 LRRs and human TLRs 19–25 LRRs. These LRRs form a continuous structure and adopt a horseshoe shape, which facilitates ligand binding (49). After ligand binding, the receptor forms an m-shaped dimer that sandwiches the ligand, bringing the cytoplasmic and transmembrane domains together to initiate signaling (50). Ligand binding causes either homodimerization or heterodimerization of TLRs and the formation of heterodimers promotes ligand diversity, whereas homodimerization increases ligand specificity (51). For example, TLR2 and TLR4 homodimerize but only the TLR4 homodimer can initiate TNFα signaling, whereas TLR1, TLR2, and TLR6 need to heterodimerize to initiate TNFα signaling (52). Differences in LRRs in the extracellular domains along with combinations of different TLRs not only promote ligand diversity to recognize a large number of PAMPs but also determine differences in the downstream signaling (53, 54).

Ligand binding then initiates a cascade of downstream signaling via the cytosolic Toll/interleukin-1 receptor (TIR) domain, which is a conserved domain shared by TLRs and the interleukin 1 receptor (IL-1R) superfamily (55). The TIR domains of TLRs dimerize upon ligand binding. This self-association leads to the recruitment of intracellular TIR-containing adaptor proteins via TIR–TIR interactions, forming a trimer. The recruitment of additional intermediates then elongates this trimer. The sequential and cooperative binding of the TIR domains amplifies the signal and results in a highly sensitive response (56). The main TIR-containing adaptor proteins are myeloid differentiation factor 88 (MyD88), Toll-interleukin 1 receptor (TIR) domain-containing adaptor protein (MAL or TIRAP), and TIR domain-containing adapter molecules (TICAM), such as TIR-domain-containing adapter-inducing interferon-β (TRIF or TICAM-1) and translocating chain-associated membrane protein (TRAM or TICAM-2). These adaptor proteins bind to TLRs and initiate major downstream signaling pathways, including nuclear factor κ-light-chain enhancer of activated B cells (NF-κB), activator protein 1 (AP-1), and interferon-regulatory factor (IRF) (56, 57).

All TLRs except TLR3 follow the MyD88-dependent pathway-activating mitogen-activated protein kinase kinase kinase 7 (MAPKKK7 or TAK1) which is downstream of TRAF6. TAK1 then leads to activation of both enzyme IκB kinase (IKK)-activating NF-κB and MAPK family c-Jun N-terminal kinases (JNK), extracellular signal-regulated kinases (ERK), and p38 leading to AP-1 activation and proinflammatory cytokine production (58). However, endosomal TLR3 and TLR4 signal via a MyD88-independent pathway involving TRIF. TLR3 associates with TRIF directly (59), whereas TLR4 associates with TRIF through TRAM (60). TRIF then activates TRAF3 activating the IRF3/7 signaling pathway leading to type 1 IFN production (61) and TRAF6 activating the NF-κB and AP-1 signaling response (62, 63).

Activation of these pathways results in the release of cytokines such as interleukin-4 (IL-4), IL-13, tumor necrosis factor-α (TNF-α), IL-1β, chemokines such as IL-8, monocyte chemoattractant protein 1 (MCP-1), macrophage inflammatory protein-1β (MIP-1β), and type I interferons (IFNs), such IFN-α and IFN-β (64). The release of the cytokines varies and depends on receptor, cell type, and species (65, 66).

**Figure 1:**
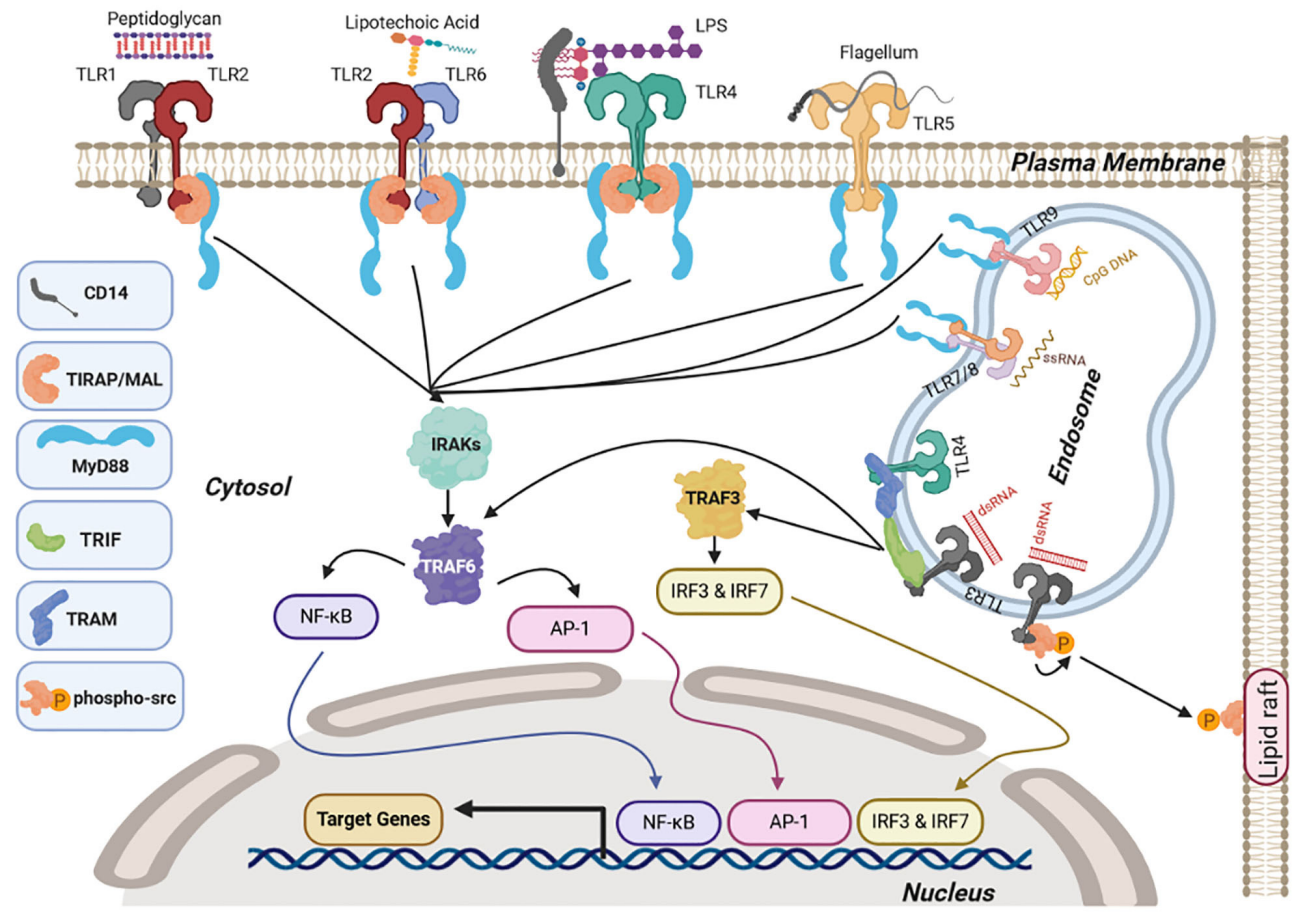
Toll-like receptor (TLR) signaling. Upon ligand binding at the cell surface, TLR1/2, TLR2/6, and TLR4 interact with myeloid differentiation factor 88 (MyD88) via the adaptor molecule Toll-interleukin 1 receptor (TIR) domain-containing adaptor protein (MAL or TIRAP) whereas TLR5 and endosomal TLRs TLR7/8 and TLR9 interact directly through MyD88. This causes activation of nuclear factor κ-light-chain enhancer of activated B cells (NF-κB) and activator protein-1 (AP-1), signaling via interleukin-1 receptor-associated kinase 4 (IRAK1 and 2) and tumor necrosis factor receptor (TNFR)-associated factor 6 TRAF6. Endosomal TLR3 and TLR4 induce NF-κb and AP-1 via TRIF associating with TRAF6 along with interferon regulatory factor IRF3/7 signaling via TRIF and TRAF3. Once NF-κB, AP-1, and IRFs are activated they translocate to the nucleus and activate transcription of target genes, including pro-inflammatory cytokines (NF-κB and Ap-1) and type I interferons (IRFs). TLR3 also induces auto-phosphorylation of the proto-oncogene Src, causing sequestration of Src in lipid rafts. Figures were drawn via biorender.com.

Not all TLRs signal via the same adaptor proteins; double-stranded RNA (ds-RNA) activation of TLR3 also initiates non-canonical signaling via recruitment and autophosphorylation of the proto-oncogene tyrosine-protein kinase Src. This leads to inhibition of cell migration, proliferation, and cell adhesion via functional sequestration of Src to lipid rafts (67). A summary of the signaling mechanisms downstream of TLRs is shown in Figure 1.

## Expression and Role of TLRs in Endothelial Cells

Endothelial cells (ECs) form the inner layer of the blood vessel, the tunica intima, and have contact to circulating cells in the lumen and vascular smooth muscle cells in the adjacent tunica media of the blood vessel. Therefore, ECs are uniquely positioned to maintain hemostasis and regulate the vascular tone via release of vasoactive mediators (68). However, ECs also respond to injury via secretion of cytokines, chemokines, and growth factors, as well as via expression of adhesion molecules (69). They hereby regulate the recruitment and activation of immune cells at the site of injury (70). ECs further recognize PAMPs and DAMPs through PRRs, particularly the TLRs (71). ECs from different species and tissues vary in the expression of TLRs. For example, human umbilical cord ECs (HUVECs) express high levels of TLR1-4, but low levels of TLR5-10, whereas human aortic ECs have high levels of all TLRs except for TLR3 and TLR9 compared to human peripheral blood mononuclear cells (PBMCs) as controls (72).

Similarly, the expression of TLRs varies between the different sections of the vascular tree, as major blood vessels have low endothelial expression of TLR2 and TLR4 (73). TLR expression is different among macrovascular and microvascular ECs. For example, baseline TLR4 levels are higher in dermal microvascular ECs than in aortic macrovascular ECs and elevated TLR4 levels correlated with increased chemokine and cytokine expression (74). In addition to mature ECs, TLRs are also found in precursor ECs, such as endothelial colony forming cells (ECFCs). ECFCs are a promising source for postnatal vascularization strategies and tissue repair (75) and are isolated from different sources (cord blood, peripheral blood, and lung) by outgrowth of EC colonies via limiting dilution from blood or tissues (76–79). ECFCs from cord blood and peripheral blood expressed mRNA for all TLRs, and umbilical cord ECFCs showed higher TLR4 expression than peripheral blood ECFCs, HUVECs, and PBMCs (80).

TLRs are also important for the differentiation and reprogramming of cells. Forced expression of a set of transcription factors is a common method to induce pluripotent stem cells. These transcription factors are octamer-binding transcription factor 4 (Oct4), sex determining region Y-box 2 (Sox2), Kruppel-like factor 4 (Klf4), and c-master regulator of cell cycle entry and proliferative metabolism (c-Myc). Lentiviral expression of these transcription factors increased the efficiency of generating induced pluripotent stem cells via TLR3/TRIF compared to non-viral expression methods (81). In addition, activation of TLR3 by its ligand double-stranded RNA (dsRNA) combined with endothelial growth factors promoted differentiation of human fibroblasts to ECs (82).

## Role of TLRs in Blood Vessel Formation and Extension

**Figure 2:**
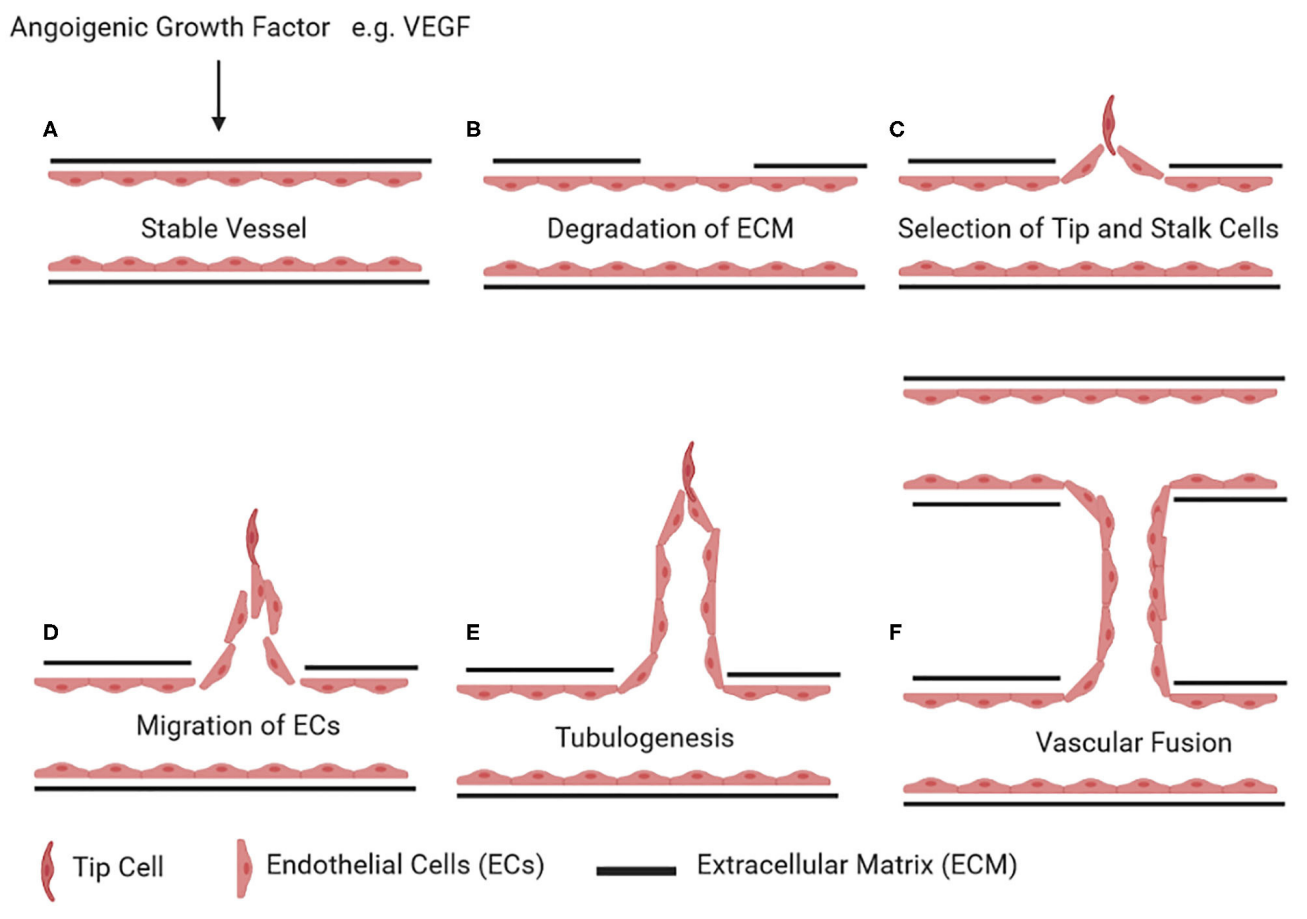
Stages of angiogenesis. Upon stimulus by an angiogenic growth factor (e.g., vascular endothelial growth factor, VEGF) on a quiescent vessel **(A)**, ECs degrade the basement membrane and the surrounding extracellular matrix **(B)**. ECs then differentiate into tip and stalk cells **(C)**. Tip cells start invading the extracellular matrix **(D)**. The stalk cells behind the tip cells continue to proliferate and form vascular tubes **(E)**. Once a tip cell fuses with the tip cell of the adjacent sprouting vessel, this results in the formation of a new connecting lumen **(F)**. Figures were drawn via biorender.com.

The network of blood vessels expands via two main mechanisms: vasculogenesis is *de novo* formation of blood vessels, and angiogenesis is sprouting or splitting of existing blood vessels (83). Sprouting angiogenesis occurs in five steps: (A) After action of angiogenic growth factor (e.g., vascular endothelial growth factor, VEGF) on a quiescent blood vessel, there is (B) degradation of the capillary basement membrane followed by (C) EC proliferation and (D) selection of tip and stalk cells. (E) ECs then migrate from the existing blood vessel to form new vascular tubes (tubulogenesis) and finally (F) connect to another blood vessel by fusion (Figure 2) (84).

TLR signaling regulates the complex process of sprouting angiogenesis at different stages of the process: The initial step of angiogenesis requires the degradation of the ECM via matrix metalloproteinases (MMPs) 2 and 9 to facilitate EC migration and tube formation (85) along with degrading endothelial tight junctions (86). Tight junctions (TJ) seal the intercellular gap to maintain endothelial barrier function, and several proteins including occludins, claudins, and zonula occludens form these tight junctions (87). The endothelial tight junction protein claudin-5 increases EC proliferation (88) but decreases EC migration and permeability, hence reducing vascular sprouting (89) similar to ECs lacking zonula occludens 1 (ZO-1), which show less migration and vascular sprouting (90). MMP9 reduced ZO-1, occludin, and claudin-5 levels via proteolysis, resulting in enhanced endothelial permeability, migration, and tube formation, and this effect was reversed with MMP9 gene silencing (86, 91). Cytokines increase MMP9 expression in an NF-κB-dependent manner via a NF-κB-binding site on the MMP9 promoter (92). TLRs promote MMP9 expression via NF-κB and extracellular signal-regulated kinase (ERK1/2) signaling pathways. TLR2 signaling influences endothelial MMP9 expression via ERK1/2 and c-Jun N-terminal kinase (JNK) signaling pathways along with MMP9-mediated reduction in tight junction proteins (93). Chlamydia pneumoniae infection promoted expression of VEGF and MMP9 in HUVECs via TLR2 and TLR4 (94). TLR4 results in EC hyperpermeability via NF-κB and AP-1-induced stromal interaction molecule 1 (STIM1) expression in human lung microvascular ECs (HLMVECs) (95). TLR2/6 activation augments EC permeability via reduced claudin-5 expression and disappearance of tight junctions, which was partly mediated by ERK1/2 (96).

In addition to tight junctions, TLRs also influence the adherens junctions between ECs. Adherens junctions are cell–cell junctions with cadherins, such as vascular endothelial (VE-) cadherin, connecting neighboring plasma membranes via homophilic interactions (97). TLR2 stimulation reduced the expression of VE-cadherin resulting in increased EC detachment and reduced EC migration while increasing MMP2/9 activity and EC apoptosis in human saphenous vein ECs (98). Similarly, in HLMVECs, TLR3 activation reduced the expression of claudin-5, ZO-1, and VE-cadherin (97).

TLR signaling further influences the expression of growth factors with a central role in regulating vascular growth. In human intestinal microvascular cells, bacterial ligands via TLRs increased angiogenic factors such as VEGF, VEGF receptor 2 (VEGFR-2), IL-8, and phosphorylation of focal adhesion kinase (p-FAK) (99). Of note, FAK promotes EC survival, angiogenesis, and vascular network stability (100). Among these growth factors, VEGF-A is a central angiogenic molecule that directs migration of endothelial tip cells during angiogenesis (101). Biglycan-stimulated TLR2 and TLR4 signaling increases VEGF-A levels, resulting in endothelial proliferation, migration, and tube formation. TLR2/4 stimulation activates NF-κB, which interacts with the hypoxia-inducible factor-1α (HIF-1α) promoter to augment HIF-1α levels. HIF-1α in turn binds to the VEGF-A, promoter increasing VEGF-A levels (102).

Activation of TLR5 by the bacterial ligand Flagellin promotes endothelial tube formation in human microvascular ECs and HUVECs through activation of the phosphoinositide 3-kinase (PI3K)/AKT1 signaling pathway (103). In rat, aortic cell culture TLR5 activation increased microvessel formation along with vessel survival but had no effect on *de novo* blood vessel formation (104).

However, TLRs also promote angiogenesis in a VEGF-independent manner. Mycoplasma lipopeptide MALP-2 in HUVECs and human monocytes/macrophages via TLR2/TLR6 promoted activation of granulocyte-macrophage colony-stimulating factor (GM-CSF). GM-CSF promoted angiogenesis in these endothelial cells while no enhanced VEGF levels in study were seen, suggesting that VEGF was not responsible for this angiogenesis in this regard (105). The end product of lipid oxidation, ω-(2-carboxyethyl)pyrrole (CEP), is generated during inflammation, wound healing, and aging. CEP interacted with TLR1/TLR2 heterodimer, promoting angiogenesis via MyD88-dependent NF-κB signaling in multiple EC lineages from human umbilical vein, mouse lung, or aorta independent of VEGF as CEP-mediated effects were unaffected by VEGFR kinase inhibition (106). In lung ECs, TLR4 signaling increases ERK-mediated activation of the transcription factor Forkhead box protein C2 (FoxC2), a transcription factor associated with lymph angiogenesis and endothelial specification by promoting delta-like 4 (DLL4) expression (107). DLL4 is a NOTCH ligand, which in angiogenic vasculature is associated with filipodia-rich endothelial tip cell formation responsible for guiding new sprouts (108).

Anti-TLR2 antibodies promoted angiogenesis in HUVECs in a manner similar to stromal cell-derived factor 1 (SDF1 also known as C–X–C motif chemokine 12 or CXCL12). This effect was mediated by Gi-protein-coupled receptor C–X–C motif chemokine receptor 4 (CXCR4) via ERK 1/2 and AKT, which was completely abolished by blocking G-protein and CXCR4, indicating a potential cross talk between TLR2 and CXCR4 pathways (109).

In contrast, TLR3 activation inhibited EC migration and tube formation in HUVECs via phosphorylation of Src independent of TRIF, TRAF3, MyD88, and IRF3 signaling (67). In primary choroidal ECs, TLR3 stimulation was anti-angiogenic and a TLR3 agonist reduced corneal neo-vascularization (34). Similarly, dsRNA of viral origin and polyinosinic:polycytidylic acid (poly(I:C)) promoted apoptosis and inhibited angiogenesis and migration in HUVECs (110). Our group has shown that TLR3 deficiency impaired migration of human pulmonary-artery ECs via caspase-3-dependent apoptosis (111). Similar to TLR3, TLR9 reduces angiogenesis, because the TLR9 agonist ODN1826 suppresses aortic angiogenesis, inhibits migration of tip cells in aortic ring assay, and suppresses corneal neovascularization *in vivo* (112). Based on these findings, activation of some TLRs appears to promote angiogenesis by downregulation of EC tight junction and adherens junction proteins, induction of angiogenic growth factors, increased proliferation, and salvation from apoptosis, but other TLR3s, such as TLR3, seem to have anti-angiogenic effects following stimulation. However, further in-depth studies are required to fully understand how signaling through the different TLRs contributes to the various stages of angiogenesis and to identify the context-dependent effects of TLR signaling on angiogenesis.

## Endothelial TLRs and Inflammation

Blood vessels are the main highway for inflammatory cells to travel to the site of injury. Inflammation and angiogenesis are therefore intricately linked processes, and signaling pathways that promote inflammation frequently also facilitate angiogenesis by inducing EC proliferation and migration. It is therefore not surprising that many proinflammatory cytokines stimulate angiogenesis, including IL-6. In mouse aortic ring assays and lung endothelial cells and HUVECs, IL-6 stimulation increased angiogenesis independent of VEGF (113). One important pathway driving IL-6 expression is the NF-κB pathway, and every member of the TLR family can signal via NF-κB (114).

TLR expression varies between different EC populations. TLR4 levels were found to be higher in microvascular ECs compared to macrovascular ECs, resulting in higher NF-κB and IL-6 levels in microvascular ECs (74). Similarly, TLR2 activation promotes in human lung microvascular EC expression of cytokines and adhesion molecules, leading to adhesion of neutrophil granulocytes (115, 116). Similar to TLR2 and TLR4, TLR9 activation causes NF-κB-mediated increase in tissue factor levels favoring a procoagulant phenotype in human coronary-artery ECs (117). In rat PAECs, TLR9 activation leads to NF-κB-mediated IL-6 production (118). In human intestinal microvascular ECs, flagellin-mediated TLR5 activation promotes expression of leukocyte adhesion molecules such as intercellular adhesion molecule 1 (ICAM-1), leading to increased transendothelial migration of leukocytes (119). TLR9 activation further enhances neutrophil adhesion on HUVECs (120). These data show that TLR signaling in ECs also promotes cross talk between ECs and inflammatory cells.

As with the foreign molecules eliciting TLR responses, the internal molecules of the body also act as DAMPs to promote immune responses via TLRs (Table 1). One of the DAMPs is high-mobility group box 1 (HMGB1). HMGB1 is an endogenous inflammatory mediator released from dying and activated cells during many pathogenic conditions including pancreatitis, cancers, atherosclerosis, and myocardial infarction (121, 122). An interesting mechanism of inflammatory signaling in ECs is the TLR-high-mobility group box 1 (HMGB1) axis. HMGB1 is an alarmin that is released in response to injury from necrotic cells, triggering an inflammatory response (123). HMGB1 signals via TLRs 2, 3, 4, 7, and 9 (26, 123, 124) to induce expression of pro-inflammatory cytokines through MyD88/NF-κB signaling (124), which in turn increases secretion of HMGB1, creating a positive feedback loop to amplify the HMGB1-mediated inflammatory effect (125). Likewise, extracellular histone-mediated activation of TLR2/TLR4 increases tissue factor expression via NF-κB and AP-1, promoting thrombus formation in human coronary artery ECs (126). TLR-mediated inflammatory signaling can also utilize G proteins. The intercellular domains of TLR2, TLR3, and TLR4 contain a consensus motif for binding of pertussis toxin-sensitive heterotrimeric G proteins Gαi/o. Gαi/o activates MAPK and Akt and interferons downstream of TLR2, TLR3, and TLR4 in ECs while having no effect on NF-κB signaling (127).

TLR 4 is the prototypical (128) and most studied TLR in literature. The functions of TLR4 as previously discussed not only are limited to its immunological role but also extend to various aspects of the vascular biology. One of the major cardiovascular disorders and the top most cause of death in the developing world is the coronary artery disease (CAD) or the ischemic heart disease (IHD), accounting for >9 million deaths globally in 2016 (129). In the heart, all the TLRs are expressed with the expression of TLR4 being the highest (130) and hence is a very important for the molecular pathogenesis of IHD. TLR4 along with TLR1 and TLR2 is highly expressed in human atherosclerotic plaques (131), with the highest expression in the shoulder regions of the plaques in the coronary arteries where the incidence of plaque rupture is highest (132). TLR4 significantly raised the levels of tissue factor (TF), a critical initiator of blood clotting from endothelial cells actively contributing to arterial thrombus formation (133). LPS-mediated TLR4 activation of human coronary artery endothelial cells resulted in increased IL-1β and TNF-α which are elevated in congestive heart failure (CHF) and CAD, hence contributing directly to their pathogenesis. Furthermore, TLR4 activation resulted in reduced cardiac function in mice whereas TLR4 deficiency promoted survival and reduction in septic shock and myocardial ischemia-induced cardiac dysfunction (134).

In addition to TLR4, endosomal TLR7 and TLR9 also play an important role in atherosclerotic lesions. Under normal conditions, major arteries of the body have a negligible expression of TLR7 and TLR9 (73). However, TLR7 levels were increased in endothelial cells, smooth muscle cells, and macrophages of mouse atherosclerotic lesions of the aortic arch (135). In apoE^*^3-Leiden mouse restenosis model, TLR7/9 activation significantly led to femoral artery cuff intimal hyperplasia and accelerated atherosclerosis and blockade of TLR7/9 significantly reduced neointima formation, atherosclerosis, and macrophage cytokine production (136). TLR7 activation may differentially respond according to disease condition. In patients with carotid endarterectomy, TLR7 was higher in atherosclerotic plaques, yet elevated TLR7 expression in the plaques was associated with better outcomes by production of anti-inflammatory cytokines (137).

RNA is another important DAMP that activates TLR3 (35, 138). We and others have shown that TLR3 expression and signaling are important in vascular biology and pulmonary hypertension (PH) (111, 139).

## TLRs in Pulmonary Hypertension

Pulmonary hypertension (PH) is a chronic, progressive disorder of the lung vasculature characterized by abnormal pulmonary-artery vasoconstriction and remodeling, leading to right heart failure and death (140). Idiopathic pulmonary arterial hypertension (iPAH) shows dysregulated EC differentiation and growth (141), and EC dysfunction is now recognized as a central process in the initiation and progression of PH (142). There are multiple aspects of EC dysfunction, which include the emergence of apoptosis-resistant, hyperproliferative ECs, dysregulated release of mediators from ECs, and endothelial-to-mesenchymal transition. One concept suggests that during development of PAH, EC apoptosis results in the selection of these apoptosis-resistant, hyperproliferative ECs. In our recent publication, we showed TLR3 deficiency in pulmonary-artery ECs from PAH patients and in lungs from a rat model of severe PH with occlusive arteriopathy (111). Treatment with the TLR3 agonist and poly(I:C) reduced severe PH and occlusive arteriopathy in rats and increased endothelial TLR3 expression in an interleukin-10 (IL-10)-dependent manner (111). Our results mirror the protective role of TLR3 signaling after balloon injury in large systemic arteries (139). However, other conflicting results seemingly contradict a protective role of TLR3 in the vasculature, but these findings may be due to a more severe degree of endothelial injury in the model system (143). Because knockout of type I IFN receptor and type I IFN treatment have frequently been associated with mostly reversible PH in highly preselected patient groups with significant comorbidities (144, 145), further evaluation of TLR3-targeted therapy is required in PAH. However, type IFN therapy seems to reduce severe angioocclusive PH in rats (146).

Recently, the HMGB1–TLR4 axis has emerged as a potential driver of pulmonary vascular remodeling in PAH. HMGB1 was elevated in concentric and plexiform lesions from patients with iPAH and in the lungs of mice exposed to chronic hypoxia (147). In addition, monocrotaline (MCT), an EC toxin causing severe PH, enhances the release of HMGB1 from injured ECs (148). Pro-inflammatory cytokines promote HMGB1 secretion, and therefore HMGB1 secretion and TLR4 may be part of a positive feedback loop enhancing inflammation and lung vascular remodeling in PAH. In addition, activation of the HMGB1/TLR4 axis in rats exposed to chronic hypoxia caused a significant decline in bone morphogenic protein receptor 2 (BMPR2), connecting HMGB1 with a well-known pathway hypomorphism in PAH pathobiology (149, 150). An inhibitor of HMGB1–TLR4 interaction has been characterized as a novel potential therapeutic, translating the findings with the TLR4–HMGB1 pathway to a potential clinical treatment in PAH, although clinical evaluation is necessary as a next step after the initial preclinical study (151, 152). Therapeutic strategies targeting TLRs in PH could be a potential avenue where both agonists and antagonists can improve pathogenesis of PH, depending on the TLR in question. In contrast to reduced PH following treatment of PH rats with the TLR3 agonist poly(I:C) (111), P5779, a blocker of the HMGB1–TLR4 interaction, reduces PH in rats (151, 152). Although other different TLR agonists and antagonists are available, there is a need for further study of TLR signaling in PH to identify additional targets depending on the role of the particular TLR in PH pathobiology.

In addition, TLR signaling can also contribute to another pathogenic mechanism in PAH, endothelial-to-mesenchymal transition (EndMT). EndMT is a process by which ECs acquire a mesenchymal cell phenotype. There is growing evidence that EndMT contributes to development and progression of pulmonary vascular remodeling and severe PAH (153–156). TLR4 activation promoted EndMT and expression of the progenitor cell marker c-kit (CD117) in mouse pulmonary ECs, indicating a potential connection between TLR4 and EndMT (157). Our group has recently shown that clonally expanded CD117^+^ ECs promote the formation of occlusive arteriopathy in rats exposed to chronic hypoxia and that CD117^+^ ECs undergo EndMT *in vitro* and *in vivo* (158). Hence, growing literature indicates that CD117^+^ ECs represent a stem-like EC population in the developing and adult lung (159, 160) and that CD117^+^ ECs contribute to lung vascular remodeling in PAH (158).

In PAH, not only ECs but also smooth muscle cells (SMCs) play a central role in the pathogenesis of the disease (161). Normal pulmonary-artery SMCs most abundantly expressed TLR3, TLR4, and TLR6. TLR3 and TLR4 co-activation resulted in IL-8 release whereas TLR3 activation alone promoted IP10 and endothelin 1 release (162). HMGB1-mediated activation of TLR4 in pulmonary-artery SMCs augmented SMC proliferation and migration along with a decline in bone morphogenic protein receptor 2 (BMPR2) signaling. Based on the dysfunctional BMPR2 pathway in PAH, it is likely that the HMGB1/TLR4 pathway has a pathogenic role in PAH (149). BMPR2-deficient mice had elevated levels of TLR4 in pulmonary-artery SMCs and lungs. LPS stimulation then leads to a significant increase in IL-6 and IL-8 production (163). These data indicate that TLR4-mediated downregulation of BMPR2 plays an auto-enhancer role driving TLR4 expression in the form of a vicious circle, which contributes to the pathogenesis of PAH. However, hypoxia reduces TLR4 expression and TLR4 knockout mice spontaneously develop PH, suggesting a protective role of TLR4 in PAH (164). This work coincided with a similar observation in ECs, which showed that hypoxia exposure reduced the expression of TLR4 and downstream nuclear translocation of AP-1 in cultured HUVECs and HPAECs (165). In stark contrast, some studies suggest that TLR4-deficient mice were protected from chronic hypoxia-induced PH (166, 167).

In summary, the existing literature indicates that TLRs have functions that exceed by far the induction of an innate immune response in ECs. Instead, TLR signaling is tightly connected with crucial elements of EC function, such as proliferation, apoptosis, angiogenic sprouting, and migration. Potential therapies targeting TLR–ligand interactions or using TLR agonists have emerged in multiple vascular diseases, in particular in PH, but careful preclinical and clinical evaluation is required when modulating TLR signaling because of the highly conserved and multifaceted effects of TLR signaling in ECs.

## Author Contributions

AB, AT, and LF conceived the manuscript. AB and LF wrote the initial draft of the manuscript. All authors edited the manuscript and approved the final version.

## Conflict of Interest

The authors declare that the research was conducted in the absence of any commercial or financial relationships that could be construed as a potential conflict of interest.
